# Supplementary material for: Future Prospects of Colorectal Cancer Screening: Characterizing Interval Cancers
Source: Cancers (Basel). 2021 Mar 16;13(6):1328. doi: 10.3390/cancers13061328 (PMC8001713; doi:10.3390/cancers13061328)
Supplement: Supplementary file 1 [file cancers-13-01328-s001.pdf]

Supplementary Materials: Future Prospects of Colorectal Cancer Screening: Characterizing Interval Cancers

Gemma Ibáñez-Sanz, Rebeca Sanz-Pamplona, Montse Garcia and on behalf of the MSIC-SC research group

Table S1. Quality assessment of Table S1 studies according to Ottawa Newcastle criteria.

Author, Year	Selection	Comparability	Outcome
Parente et al., 2013[37]	***	*	*
Shin et al., 2013[38]	****	*	***
Chiu et al., 2015[39]	****	*	***
Jensen et al., 2016[40]	***	*	***
Giorgi Rossi et al., 2017[41]	***	*	**
Portillo et al., 2017[42]	****	*	***
van der Vlugt et al., 2017[43]	****	*	***
Burón et al., 2018[44]	****	*	***
Novak Mlakar et al., 2018[45]	****	*	***
van der Veerdonk et al., 2019[46]	****	*	*
Toes-Zoutendijk et al., 2020[47]	****	*	***
Zorzi et al., 2020[48]	****	*	***

A study can be awarded a maximum of one star for each numbered item within the Selection (*n* = 4) and Outcome (*n* = 2) categories. A maximum of two stars can be given for Comparability.

Table S2. Quality assessment of Table 2 studies according to Ottawa Newcastle criteria.

Author, Year	Selection	Comparability	Outcome
Sawhney et al., 2006[76]	***	**	***
Shaukat et al., 2010	***	**	***
Arain et al., 2010[77]	***	**	***
Shaukat et al., 2010[78]<br>Shaukat et al., 2012[79]	***	**	***
Nishihara et al., 2013[75]	***	*	***
Richter et al., 2014[80]	****	*	***
Cisyk et al., 2015[81]	****	**	***
Lee et al., 2016[83]	***	*	***
Stoffel et al., 2016[15]	****	*	***
Walsh et al., 2016[49]	****	**	***
Cisyk et al., 2018[82]	****	**	***
Samadder et al., 2019[84]	****	**	***
Tanaka et al., 2020[85]	***	-	***

A study can be awarded a maximum of one star for each numbered item within the Selection (*n* = 4) and Outcome (*n* = 2) categories. A maximum of two stars can be given for Comparability

© 2021 by the authors. Licensee MDPI, Basel, Switzerland. This article is an open access article distributed under the terms and conditions of the Creative Commons Attribution (CC BY) license (http://creativecommons.org/licenses/by/4.0/).
